# A meta-analysis of ferric carboxymaltose versus other intravenous iron preparations for the management of iron deficiency anemia during pregnancy

**DOI:** 10.61622/rbgo/2024AO21

**Published:** 2024-03-15

**Authors:** Sanjay Gupte, Ashis Mukhopadhyay, Manju Puri, P. M. Gopinath, Reena Wani, J. B. Sharma, Onkar C. Swami

**Affiliations:** 1 Gupte Hospital & Center for Research in Reproduction Department of Obstetrics and Gynecology India Department of Obstetrics and Gynecology, Gupte Hospital & Center for Research in Reproduction, India.; 2 CSS College of Obstetrics Gynae. & Child health Department of Gynecology Kolkata India Department of Gynecology, CSS College of Obstetrics, Gynae. & Child health, Kolkata, India.; 3 Lady Hardinge Medical College Department of Obstetrics and Gynecology New Delhi India Department of Obstetrics and Gynecology, Lady Hardinge Medical College, New Delhi, India.; 4 Institute of Obg & IVF SIMS Hospital Department of Obstetrics and Gynecology Vadapalani Chennai India Department of Obstetrics and Gynecology, Institute of Obg & IVF SIMS Hospital, Vadapalani, Chennai, India.; 5 HBTMC & Dr RN Cooper Hospital Department of Obstetrics and Gynecology Mumbai India Department of Obstetrics and Gynecology, HBTMC & Dr RN Cooper Hospital, Mumbai, India.; 6 Department of Obstetrics and Gynecology AIIMS New Delhi India Department of Obstetrics and Gynecology, AIIMS, New Delhi, India.; 7 Emcure Pharmaceuticals Ltd Pune India Emcure Pharmaceuticals Ltd, Pune, India.

**Keywords:** Ferric carboximaltose, Iron sucrose, Iron polymaltose, Intravenous iron, Anemia, Iron-deficiency anemia, Pregnancy, Hemoglobin, Ferritin, Ferric oxide, saccharated

## Abstract

**Objective:**

We conducted a meta-analysis of randomized clinical trials evaluating the clinical effects of ferric carboxymaltose therapy compared to other intravenous iron in improving hemoglobin and serum ferritin in pregnant women. We also assessed the safety of ferric carboxymaltose vs. other intravenous iron.

**Data source:**

EMBASE, PubMed, and Web of Science were searched for trials related to ferric carboxymaltose in pregnant women, published between 2005 and 2021. We also reviewed articles from google scholar. The keywords "ferric carboxymaltose," "FCM," "intravenous," "randomized," "pregnancy," "quality of life," and "neonatal outcomes" were used to search the literature. The search was limited to pregnant women.

**Selection of studies:**

Studies related to ferric carboxymaltose in pregnancy were scanned. Observational studies, review articles, and case reports were excluded. Randomized studies in pregnant women involving ferric carboxymaltose and other intravenous iron formulations were shortlisted. Of 256 studies, nine randomized control trials were selected.

**Data collection:**

Two reviewers independently extracted data from nine selected trials

**Data synthesis:**

The final effect size for increase in hemoglobin after treatment was significant for ferric carboxymaltose vs. iron sucrose/iron polymaltose (standard mean difference 0.89g/dl [95% confidence interval 0.27,1.51]). The final effect size for the increase in ferritin after treatment was more for ferric carboxymaltose vs. iron sucrose/iron polymaltose (standard mean difference 22.53µg/L [-7.26, 52.33]). No serious adverse events were reported with ferric carboxymaltose or other intravenous iron.

**Conclusion:**

Ferric carboxymaltose demonstrated better efficacy than other intravenous iron in increasing hemoglobin and ferritin levels in treating iron deficiency anemia in pregnant women.

## Introduction

Iron deficiency anemia (IDA) is a commonly noted public health nutritional problem in pregnant women. Nearly one-third of women of reproductive age are anemic. The World Health Organization estimates that 40% of pregnant women are anemic.^(1)^ A comprehensive systematic review from 107 countries reported the prevalence of anemia in pregnant women as 38% (95% confidence interval [CI] 33–43%), accounting for 32 million pregnant women.^(2)^ According to the World Health Organization, the prevalence of anemia in pregnant women in India was 50.1%. Compared to 2000 data, the prevalence of anemia in pregnant women reduced by merely 3.6% in 2019.^(3)^ Nearly 75% of anemia in pregnant women manifests as iron deficiency anemia (IDA).^(4)^ The prevalence of iron deficiency (ID) in pregnant women in India is the highest globally.^(5)^

Hemoglobin levels of less than 11 g/dL during pregnancy are considered out of the normal range.^(6)^ According to the World Health Organization, the hemoglobin level of 10–10.9 g/dL is defined as mild, 7–9.9 g/dL as moderate, and <7 g/dL as severe anemia in pregnant women.^(5)^

Iron requirements in pregnant women are greater than in non-pregnant women primarily to meet the expanding maternal red blood cell mass, fetal iron requirement, and blood loss during delivery.^(7)^ If not managed, ID can cause adverse perinatal consequences to the mother and the fetus—preterm labor, premature rupture of membranes, perinatal blood transfusions, intensive care unit (ICU) admissions, postpartum depression, small-for-gestational-age infants, and increased maternal and fetal mortality.^(8–10)^

The choice to treat IDA depends on the cause and severity of ID. Oral iron is usually the first line of treatment. Several months of oral treatment are required to meet the clinical requirement.^(11)^ Generally, IV iron is considered when the treatment response is inadequate with oral formulations. IV iron is preferred in case of low iron absorption due to intestinal disease, intolerance of oral iron, non-compliance to oral iron supplementation, or the need for rapid and adequate treatment in the event of bleeding.^(12)^ IV iron enables rapid correction of anemia and its symptoms.^(13,14)^ Ferric carboxymaltose (FCM), iron sucrose (IS), and iron polymaltose (IP) are newer IV iron formulations used to treat IDA.

Ferric carboxymaltose is a novel colloidal solution that comprises a ferric hydroxide core stabilized by a carbohydrate shell. The colloidal formulation facilitates the controlled delivery of iron to target tissues with minimal risk of releasing large amounts of ionic iron into the serum. FCM is a non-dextran IV iron agent with inherently a very low immunogenic profile and therefore it is less likely to cause anaphylactic reactions. The pharmacological properties of FCM facilitates administration of large doses (maximum of 1000 mg/infusion) by single administration (15-minute infusion) without a mandatory test dose.^(15)^

Iron sucrose is available as a solution for injection or concentrate for solution for infusion. Iron in IS is composed of a polynuclear iron(III)-hydroxide core enclosed within a large number of non-covalently bound sucrose molecules (ligand).^(13)^ The efficacy of IS in treating anemia is well-established. However, it warrants multiple dosing and hence is associated with low compliance.^(15)^ In IP, the iron(III)-polymaltose complex polynuclear iron(III)-hydroxide is surrounded by polymaltose ligands.^(13,14)^ IP must be given as an intermittent IV infusion. Up to 2500 mg of elemental iron is infused for approximately three to five hours.^(16)^

In pregnant women with mild (10-10.9 g/dL) to moderate (7-9.9 g/dL) anemia, parenteral iron (IV FCM or IS) may be considered as the first line of management in pregnant women who are detected to be anemic late in pregnancy, in whom compliance is likely to be low (high chance of lost to follow-up), or there is no improvement with oral iron.^(17)^ Pregnant women should be managed with IV IS or FCM in a hospital setting. Pregnant women with severe IDA (Hb: 5.0-6.9 g/dL) should be managed with IV FCM or IS.^(17)^ One of the disadvantages of IV IS is that it requires multiple visits to administer the required dose as the maximum dose allowed per week is limited.^(18)^

Clinical trials have confirmed the effectiveness of FCM in rapidly improving anemia and replenishing iron stores.^(19–22)^ FCM has been used in several clinical settings of anemia, including gynecological and obstetric conditions, nondialysis-dependent chronic renal failure, inflammatory bowel disease, heart failure, chemotherapy-associated anemia without concomitant use of erythropoiesis-stimulating agents, and nonresponse to oral iron.^(23–28)^ Health-related quality-of-life (HR-QOL) improved with FCM.^(19)^ Ferric carboxymaltose exhibits a good tolerance profile in patients with IDA. Most drug-related adverse events with FCM were mild to moderate in severity and did not differ significantly from oral iron.^(19)^

Several clinical trials have evaluated the safety and efficacy of FCM. A few meta-analyses have evaluated the effect of FCM in treating IDA. Ferric carboxymaltose was better than other IV iron preparations, according to a recent meta-analysis of studies in women with IDA due to gynecological and obstetric conditions.^(24)^ However, the meta-analysis was limited by the small number of RCTs and high heterogeneity.^(24)^ There is no exclusive meta-analysis of randomized control trials (RCTs) comparing FCM with other IV iron formulations in treatment of IDA during pregnancy.

We conducted a meta-analysis of randomized clinical trials to evaluate the clinical effects of FCM therapy vs. other IV iron formulations in improving hemoglobin and serum ferritin in pregnant women. We also assessed the safety of FCM vs. other IV irons.

## Methods

We conducted a meta-analysis of randomized clinical trials evaluating the clinical effects of FCM therapy on hemoglobin and serum ferritin levels in pregnant women, neonatal outcomes, and patient-reported outcomes. We included RCTs conducted in pregnant women with IDA treated with IV FCM or other IV formulations (IS or IP). We included studies that enrolled women with 12-36 weeks gestational age, baseline hemoglobin of 6.0-10.9 g/dL, or serum ferritin of <100 mcg/L (100 ng/mL). We excluded studies that compared IV FCM with any other oral preparations. Our primary outcome was a mean increase in hemoglobin and serum ferritin levels. The secondary outcomes were neonatal (birth weight, mortality indicators, Apgar score, and need for hospitalization), patient-reported outcomes (improvement in fatigue scores or quality of life), and safety of FCM in treating IDA during pregnancy. This study did not require the ethics committee’s approval because all analyses were based on previously published RCTs.

Two independent researchers systematically searched indexed journals in EMBASE, PubMed, and Web of Science between 2005 and 2021. We also reviewed articles from google scholar. We used the following string "ferric carboxymaltose" AND "pregnancy" to fetch all articles and manually filtered RCTs comparing FCM with IS or IP.

Studies related to FCM and pregnancy were scanned. Observational studies, review articles, and case reports were excluded. Randomized studies in pregnant women involving FCM and other IV iron formulations were shortlisted. The full text of shortlisted articles was reviewed, and data were compiled.

We formulated a data collection form before the literature search strategy. Independent reviewers extracted all relevant data in an excel sheet. Fulltext articles were retrieved, and data was populated in the excel sheet with parameters viz: author information, year of publication, study setting, sampling strategy, sample size, mean hemoglobin (pre and post-intervention), the mean increase in hemoglobin, mean ferritin (pre and post-intervention), and a mean increase in ferritin. We also recorded any neonatal or patient-reported outcomes.

RevMan5.4 was used to obtain the risk of bias of each included study across the following eight domains: sequence generation, allocation concealment, blinding of participants and personnel, blinding of outcome assessment, incomplete outcome data, selective outcome reporting, other biases, and overall risk of bias assuming a wide variation in the data. We used a random-effects meta-analysis for combining data. Statistical analyses were performed using RevMan 5.4. Mean differences (MD) with 95% CIs were calculated for continuous variables. We conducted sensitivity analyses examining effects by removing studies with a high risk of bias.

The mean difference or standard mean difference for hemoglobin and serum ferritin levels was calculated or collated from the studies. Few studies reported endpoint data, and others reported changes from baseline data (standard deviations). All data were combined using the mean difference (MD) as the common scale. Where key data (standard deviations) were missing, we used methods recommended in the Cochrane Handbook for Systematic Reviews of Interventions to calculate them.^(29)^ First, we calculated the t-value from a P-value, estimated the standard error, and obtained the standard deviation. We used the I² statistic, Tau², and Chi² test for heterogeneity to quantify the level of heterogeneity among the trials in each meta-analysis.

## Results

### Literature search and patient population

**Figure 1:**
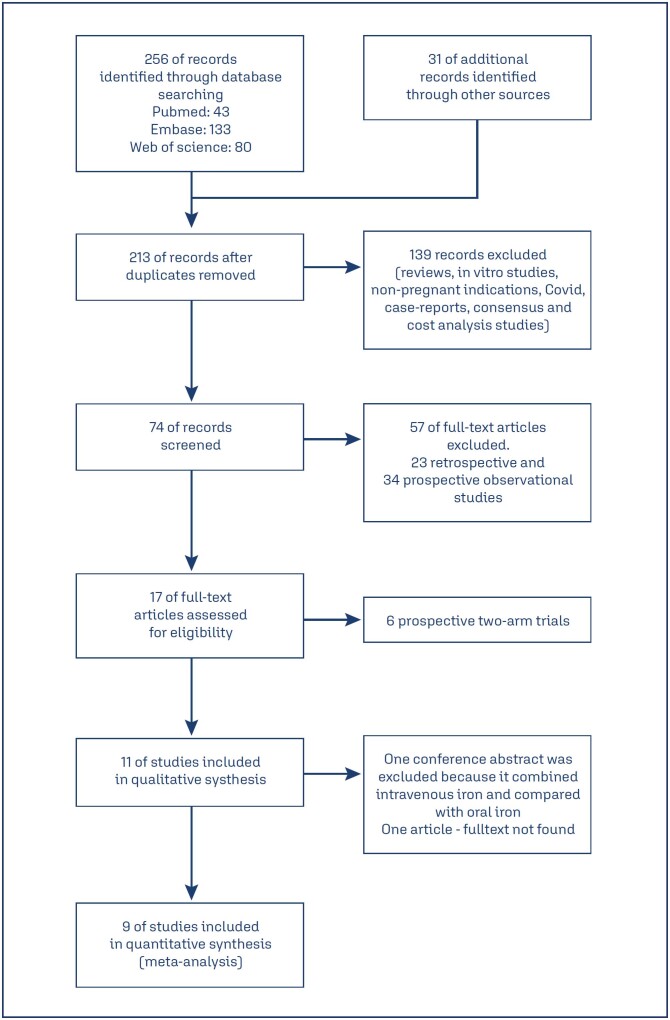
PRISMA method of searching articles

Of 256 articles, nine were included using the PRISMA method (Figure 1).^(18,30–36)^ A total of 256 studies were obtained from Pubmed (n=43), Embase (133) and Web of Science (n=80). Thirty-one studies were obtained from a direct search google/google scholar. Thirty-three duplicate studies and 139 studies (review articles, in vitro, non-pregnant indications, Covid, case reports, consensus documents and cost-analysis studies) were excluded. Of 74 studies, 63 (retrospective and prospective observational studies) were excluded. Finally, 11 studies were selected, but one was a conference abstract, and one full-text article was unavailable. Hence nine studies were included in the final analysis. Of 1406 pregnant women who received IV iron injections, 613 received FCM, 530 received IS, and 82 IP. The age ranged from 19 years to more than 42 years. The gestational age was >14 weeks.

### Assessment for the risk of bias

**Figure 2:**
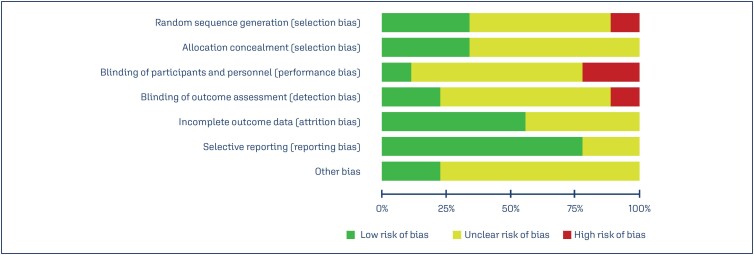
Risk of bias of studies included in the meta-analysis

**Figure 3:**
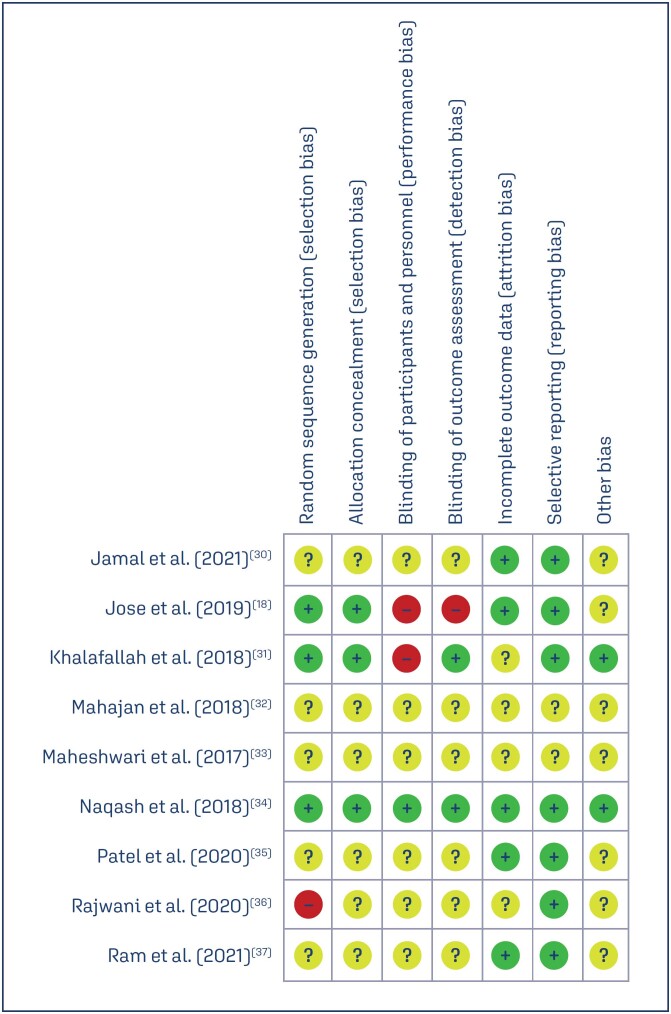
The quality of studies included in the meta-analysis

The risk of bias in studies included in the meta-analysis is shown in figures 2 and 3. Except for three studies,^(18,31,34)^ all other studies did not describe the randomization and allocation concealment method. Most of the studies did not provide details of selection, method of randomization and allocation, and blinding of participants.

### Change in hemoglobin levels

**Figure 4:**
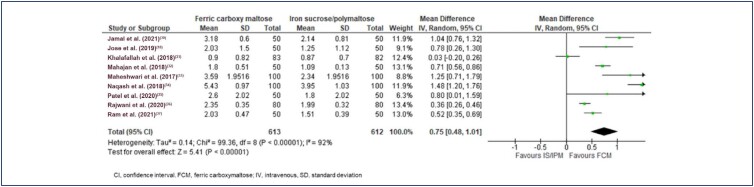
Forest plot of serum hemoglobin levels (g/dL), FCM vs. other IV iron

**Figure 5:**
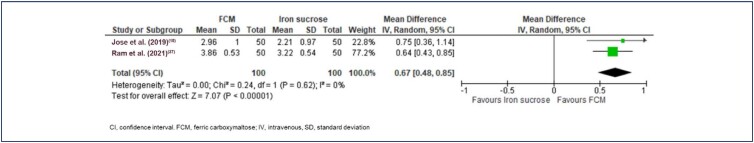
Forest plot of serum hemoglobin levels (g/dL) at 12 weeks, FCM vs. iron sucrose

**Figure 6:**
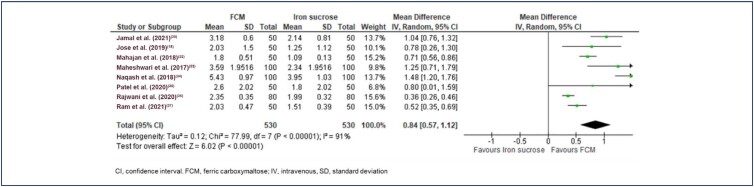
Forest plot of serum hemoglobin levels (g/dL) FCM vs. iron sucrose

There was variation in the outcome time points; hence, studies reporting post-interventional outcomes at 3 and 4 weeks combined in the meta-analysis. The average baseline hemoglobin levels were 8.69 g/dL, 8.36 g/dL, and 11.4 g/dL in the FCM, IS, and IP groups. Khalafallah et al.^(31)^ study is the only study that compared FCM with IP, and the study included patients with hemoglobin levels ≥8.5 g/dL but ≤12 g/dL. The final effect size for an increase in hemoglobin after treatment was significant for FCM vs. other IV iron (MD 0.75 g/dL [95% CI 0.48 to 1.01] heterogeneity: Tau² = 0.14 Chi² = 99.36, df = 8 (p<0.00001); I²=92%; p<0.00001; (Figure 4). Two studies, Jose et al.^(18)^ and Ram et al.,^(37)^ assessed the effect of treatment at 12 weeks, and the change in hemoglobin levels was significantly greater in FCM vs. IV IS (MD: 0.7 [95% CI 0.48 to 0.85] Tau² = 0.00 Chi² = 0.24, df = 1 (p=0.62); I^2^=0%; p<0.00001; (Figure 5).^(18,37)^ In eight studies, FCM was compared against IS and the difference in treatment effect was in favor of FCM (MD: 0.84 [95%CI 0.57, 1.12] Tau² = 0.12 Chi² = 77.99, df = 7 (p<0.00001); I^2^=91%; p<0.00001; (Figure 6).^(18,30,32–37)^ A sensitivity analysis without including Jose et al.^(18)^ was also in favor of FCM vs. other IV iron preparations (MD 0.74 [95% CI 0.46 to 1.03] Tau² = 0.14 Chi² = 98.63, df = 7 (p<0.00001); I^2^=93%; p<0.00001).

### Change in serum ferritin levels

**Figure 7:**
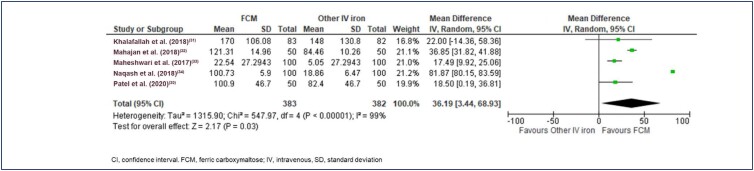
Forest plot of serum ferritin (µg/L), FCM vs. other IV iron

**Figure 8:**
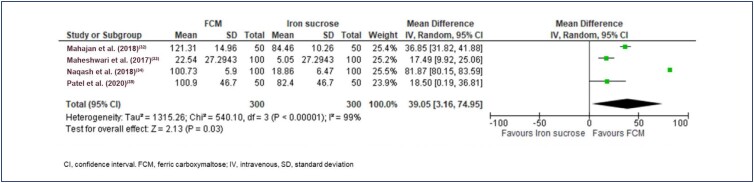
Forest plot of serum ferritin (µg/L), FCM vs. Iron sucrose

Jamal et al.,^(30)^ Rajwani et al.,^(36)^ and Ram et al.,^(37)^ did not provide data on serum ferritin levels. Jose et al.^(18)^ provided the median value and hence was excluded from the analysis. The average baseline ferritin levels were 21.13 µg/L, 18.84 µg/L, and 13.00 µg/L in the FCM, IS, and IP groups, respectively. The final effect size for an increase in ferritin after treatment was in favor of FCM vs. other IV iron (MD 36.19 [95% CI 3.44 to 68.93]; Tau² = 1315.90, Chi² = 547.97, df = 4 (p<0.00001); I^2^=99%; p=0.03) (Figure 7). The final effect size for an increase in ferritin after treatment was in favor of FCM vs. IS (MD 39.05[95% CI 3.16 to 74.95]; Tau² = 1315.26, Chi² = 540.10, df = 3 (p<0.00001); I^2^=99%; p=0.03; (Figure 8). In the Jose et al.^(18)^ study, the median difference in serum ferritin from baseline at 3, 6, and 12 weeks were 335.1 µg/L, 283.6 µg/L, and 179.6 µg/L, respectively. In comparison, the difference in the IS group was 289 µg/L, 224.3 µg/L, and 136.5 µg/L, respectively, at weeks 3, 6, and 12 weeks.

### Neonatal outcomes

Neonatal complications were reported in one study by Khalafallah et al.^(31)^ wherein treatment with iron infusions did not affect the fetal outcomes (fetal outcomes in terms of Apgar scores at 1, 5, and 10 minutes, weight, length, or head circumference of the baby, neonatal resuscitation, or complications). The APGAR was 9 at 1 and 5 minutes in infants born to women treated with FCM or IS. The incidence of neonatal complications (11 [13.8%] vs. 7 [9.6%]) and neonatal resuscitation (17 (21.3% vs. 11 (15.1%; p=0,4038) was numerically high in the FCM vs. IS, but were not statistically significant, One and four patients in the FCM and IS group had threatened premature labor (p=0.6066). There was no statistically significant correlation between different iron supplements and pregnancy complications. The association between postnatal and postpartum outcomes for different iron supplements was statistically insignificant. Cord blood ferritin and hemoglobin levels in FCM were 187.4 µg/L and 15.7 g/dL, and in the IS group, it was 214.1 4 µg/L and 16.26 g/dL.^(31)^ In the Jose et al.^(18)^ study, seven patients treated with FCM delivered premature infants before 37 weeks of gestation. One infant was delivered prematurely within two weeks of the last dose. Six patients treated with IS delivered preterm. One patient who received treatment from 17 weeks of gestation had a spontaneous abortion at 22 weeks. No statistical difference in birth weight was seen between FCM and IS groups (2834.1 g vs. 2864.7 g; p=0.73).^(18)^

### Patient-reported outcomes

The effect of FCM on quality of life was reported by Naqash et al.^(34)^ and Khalafallah et al.^(31)^ As there were no other data, we could not conduct a meta-analysis. In the Naqash et al.^(34)^ study, the quality of life of pregnant women was better in those treated with FCM, and women were satisfied with the FCM treatment. Treatment with FCM resulted in fewer hospital visits and minimized the utilization of hospital resources compared to IS.^(31,34)^ Khalafallah et al.^(31)^ assessed the correlation between the quality of life and serum ferritin levels. Restoring ferritin levels to >30 µg/L improved quality of life.^(31)^ This effect was evident with FCM or IP. Ninety-three percent of women who received FCM or IP achieved a ferritin level of >30 µg/L four weeks after treatment.^(31)^ Intravenous FCM was reportedly more convenient than IP because of shorter duration of administration (15 min vs. 2 h).^(31)^

### Safety and tolerability

Adverse events were described in seven studies (Chart 1). In the Maheshwari et al.^(33)^ study, IV IS and FCM were safe. In the Naqash et al.^(34)^ study, there were no reports of serious adverse events. Only one patient complained of a headache after the second dose of FCM. Iron sucrose was associated with nausea, tingling, headache, and arthralgia.^(33)^ There were no reports of serious adverse events in the FCM and IS group in the Jose et al.^(18)^ study. One and two patients, respectively, treated with FCM and IS experienced injection site reactions.^(18)^ There were no serious adverse reactions or complications reported FCM or IP in Khalafallah et al.^(31)^ study. Flu-like symptoms with bone ache and nausea were reported in 6.3% and 11.5% of the patients treated with FCM and IP, respectively.^(31)^ In the Mahajan et al.^(32)^ study, mild adverse reactions were observed in 30% and 48% of patients treated with FCM and IS, respectively. In the Patel et al.^(35)^ study, three and four women, treated with FCM and IS experienced mild local reactions. One woman had a severe anaphylactic reaction with a respiratory problem following IS infusion.^(35)^ Rajwani et al.^(36)^ did not find any serious adverse effect with either FCM or IS.

**Chart 1:** Relative adverse events reported in the studies included

	Jose et al.^(18)^		Khalafallah et al.^(31)^		Mahajan et al.^(32)^		Maheshwari et al.^(33)^		Naqash et al.^(34)^		Patel et al.^(35)^		Rajwani et al.^(36)^	
	FCMn	IS,n	FCM n (%)	IS n (%)	FCMn (%)	IS n(%)	FCM n (%)	IS n(%)	FCMn	ISn	FCMn	ISn	FCMn	ISn
Serious adverse events	0	0	0	0	0	0	0	0	0	0			0	0
Injection site reaction	1	4			1 (2%)	3 (6%)					3	4		
Mild epigastric pain	0	2												
Elevated serum transaminases	1	0												
Hypophosphatemia	2	3												
Flu-like symptoms with bone ache and nausea			5 (6.3%)	9 (11.5%)										
Delayed reactions			1	3										
Diarrhea					2 (4%)	5 (10%)								
Nausea					3 (6%)	3 (6%)	10 (13.6%)	1 (1.32%)	0	3				
Constipation					3 (6%)	3 (6%)								
Abdominal pain					0	3 (6%)	1 (1.32%)	2 (2.63%)						
Headache					2 (4%)	2 (4%)	1 (1.32%)	5 (6.58%)	1	2				
Dysgeusia					0	2 (4%)								
Skin discoloration					1(2%)	2 (4%)								
Vomiting					2 (4%)	1(2%)	2 (2.63%)	1 (1.32%)						
Hot flushing					1(2%)	0	2 (2.63%)	3 (3.95%)						
Heartburn							3 (3.95%)	4 (5.26%)						
Dyspepsia							0	4 (5.26%)						
Black stools							3 (3.95%)	4 (5.26%)						
Arthralgia									0	1				
Severe anaphylactic											0	1		

## Discussion

Iron requirements in pregnant women are greater than in non-pregnant women. Pregnant women require nearly 4.4 mg/day of iron. The iron requirement during the first trimester is 0.8 mg/day, between 4 and 5 mg/day in the second trimester, and about 6 mg/day in the third trimester, equivalent to the total requirement of around 1000 to 1240 mg in a singleton pregnancy.^(7,38,39)^ FCM can be administered in a single large dose of 1000 mg over a short duration of 15 minutes which fulfills the total iron requirement in the majority of pregnant women.^(15,17)^ Iron sucrose requires multiple infusions over a few days to meet the same requirement, and an infusion of IP to fulfil the iron requirement would take 4 to 5 hours.^16^ Therefore, may be preferred over these iron supplements in pregnancy.^(15,18)^

Our meta-analysis demonstrated that improvement in hemoglobin with FCM was significant compared to other IV iron. There was a clinically significant improvement in serum ferritin with FCM versus other IV irons (IS/IP), but this difference did not reach clinical significance. The improvement in hemoglobin with FCM is evident from 2 weeks onwards. In pregnant women, rapid correction of ID is warranted, especially in those with bleeding due to placenta previa or those with IDA in the third trimester. Clinical trials and real-world experience have provided significant evidence on the efficacy of FCM in rapidly correcting ID and IDA due to various etiologies.^(19,21,40,41)^

With increasing odds of preterm labor in mothers with IDA, the iron reserve is probably limited in preterm infants as the fetal iron reserve increases with fetal liver growth, which is optimal from 32 weeks onwards. More than two-thirds of the neonatal reserve is attained from the third trimester.^(42)^ Hence, it is obvious that gestational age and maternal ID influence infants’ iron reserve. Up to the first six months, infants largely depend on the iron stores acquired during pregnancy. Preterm infants and small for gestational-age infants are likely to have low reserves of iron during an early age. The Valencia Infant Anaemia Cohort (VIAC) study showed that infants born to mothers with ID were nearly seven times more likely to have ID during their first year than those born to mothers without ID.^(43)^ One of the advantages of FCM is the rapid replenishment of iron reserves and improvement in hemoglobin. In our meta-analysis, we assessed the effect of FCM and other IV iron at 3 or 4 weeks after treatment.

There was a significant impact on improvement in hemoglobin levels at 3 or 4 weeks. Improvement in ferritin with FCM did not reach significance, perhaps due to fewer studies reporting the effect of FCM on ferritin. In the Rajwani et al.^(36)^ study, the hemoglobin levels improved by 1.06±0.21 g/dL within a week of administering FCM compared to 0.95±0.41 g/dL with IS/IP. Naqash et al.^(34)^ and Mahajan et al.^(32)^ reported the treatment effect 2 weeks after intervention and found a significant improvement in the hematological profile.^(32,34)^ Further, the effects of FCM were sustained for a longer duration of up to six and 12 weeks. The long-term treatment effect of FCM was evident until 12 weeks.^(18)^

Shin et al.^(24)^ conducted a meta-analysis to assess the safety and efficacy of FCM and IS in obstetric and gynecologic patients with IDA. IV FCM was superior to IS as an iron replacement option to treat IDA in women with obstetric and gynecologic conditions. With FCM, patients achieved higher ferritin compared to IS (mean difference, 24.41ng/mL; 95% CI, 12.06–36.76; p=0.0001). Similarly, hemoglobin levels were significantly higher among patients who received FCM than those who received IS (mean difference, 0.67; 95% CI, 0.25–1.08; p=0.002).^(24)^

Qassim et al.^(44)^ conducted a systematic review of the safety and efficacy of IV IS, IP, and FCM in pregnancy. The systematic review did not find any significant impact of IV iron on fetal or neonatal outcomes as the number of neonates in the only study by Khalafallah et al.^(31)^ was very small to draw any conclusion. Nevertheless, the review reiterated the improvement in hematological parameters in pregnant women with IV iron. The median prevalence of adverse events was lower with FCM than with IP.^(44)^ The review noted that the median prevalence of adverse events was lower with IP than FCM and IS. However, moderate or severe adverse reactions reported were lower with FCM compared to IP and IS. In the meta-analysis by Shin et al.,^(24)^ the incidence of adverse events in the FCM group was lower by 47% compared to the IS group (RR, 0.53; 95% CI, 0.35–0.80; I2 = 0%; p=0.003). Ferric carboxymaltose is generally well tolerated, with a low risk of hypersensitivity reactions.^(21,22)^ There were no significant differences in the adverse events in all the studies included in the meta-analysis. Most of the studies reported mild events. We did not perform a meta-analysis because not all studies included had quantitative reports of adverse events, and no statistical differences between the IV formulations could be achieved.

Our meta-analysis found that hemoglobin and ferritin levels significantly improved with FCM compared to IS or IP. There were no serious safety and tolerability issues with FCM. Hence, it is prudent to consider FCM in pregnant women with IDA.

Rapid administration of a couple of doses of iron is likely to increase patients’ compliance, and FCM is formulated in such a way that it could deliver large doses of iron in a shorter time.^(15,45)^ Hence, an ultra-short duration of treatment with FCM is one of the favorable characteristics and hence advantageous over the conventional IV iron.^(46)^

Except for Khalafallah et al.,^(31)^ all other studies did not provide a precise method of randomization or concealment. Overall, the risk of bias was not low in all the included studies. Change in ferritin from baseline was not provided in all the studies included; hence, a more precise pooled estimate could not be found. We could derive the exact doses of FCM or other IV iron from achieving a clinical effect from the studies included. Our meta-analysis included a small number of RCTs with high heterogeneity. The studies included did not classify anemia (mild, moderate or severe), and the outcomes were generally reported for anemia. There was a disparity between the studies regarding gestational age. The outcomes could not be grouped according to the gestation age as early, mid or late pregnancy. Further, we did not find information regarding co-morbidities such as placenta previa, postpartum hemorrhage, early bleeding during pregnancy, or bleeding disorders regardless of pregnancy. A quantitative analysis of secondary endpoints (neonatal outcomes, maternal outcomes, patient-reported outcomes, quality of life, and safety) was not feasible owing to a lack of data and the number of participants from only the study by Khalafallah et al.^(31)^ was very small to draw any conclusion. Therefore, the results should be interpreted considering the above limitation. Another limitation of this meta-analysis is the lack of comparison of cost between the treatments. Our meta-analysis consolidates the efficacy of FCM vs. other IV iron in treating IDA in pregnancy.

## Conclusion

Ferric carboxymaltose demonstrated better efficacy than other intravenous iron in increasing hemoglobin and ferritin levels in treating IDA in pregnant women. Ferric carboxymaltose helps in rapidly correcting ID without a significant compromise on safety and tolerability. A shorter duration of FCM administration is more convenient than other IV iron. Iron content could be replenished with a single FCM injection or, as clinically deemed necessary in pregnant women with IDA. Further large-scale studies are warranted to confirm the effects of FCM on ferritin levels in pregnant women with IDA.
